# Gamifying the Infection Control Curriculum: The Impact on Nursing Students’ Knowledge, Exam Performance, and Course Perception

**DOI:** 10.1017/ash.2021.128

**Published:** 2021-07-29

**Authors:** Tomislav Mestrovic, Jasminka Talapko, Tina Cikac, Marijana Neuberg

## Abstract

**Background:** Unlike passive didactic teaching, the introduction of innovative active-learning approaches to university nursing curricula aims to address the educational content in an interactive learning environment, improving in turn the learning process and problem-solving skills indispensable for future infection control professionals. One such strategy is the use of educational games, which can motivate students and enhance the degree of their engagement. We appraised the effectiveness of introducing an interactive game based on a popular television quiz show “Who Wants to be a Millionaire?” for educational attainment, exam performance, and course perception in nursing students. **Methods:** A whole generation of second-year undergraduate nursing students (126 female and 27 male participants; age range, 19–41 years) from a public university in Croatia (University Centre Varazdin, University North) were divided into 2 groups by cluster randomization; one group had received additional hours of game play after core training curriculum in a “Hygiene and Epidemiology” course, while the other had not. Game play was accomplished by employing ‘edutaining’ interactive multimedia approach, and covered primarily hand hygiene, cough etiquette, the use of personal protective equipment, sterilization and disinfection, and safe injection practices. Quantitative results of multiple-choice exams were used to evaluate any differences in the knowledge level of respective groups. A satisfaction opinion survey was used to gauge attitudes of students attending the course. Statistical significance was defined as *P* < .05 (2-tailed). **Results:** The mean baseline examination score was 28.30±5.79 points for the game group and 24.65±5.94 points for the control group, demonstrating improved knowledge retention when the interactive game was introduced into the curriculum. The statistically significant improvement in knowledge was observed in the domains of personal protective equipment and safe injection practices. There was no statistically significant difference in the overall scores between male and female students. Students who were subjected to game play expressed more agreement on a Likert scale regarding course enjoyment and innovativeness, albeit they did not differ from control group when assessing the educational merit of the course. **Conclusions:** Introducing interactive games to university courses that cover infection control may boost student enjoyment and enhance long-term retention of information, as confirmed by this study. Nonetheless, extra care should be taken when specific games that have not been assessed objectively are implemented. Further research in this field will elucidate how this increased knowledge retention in infection control principles translates to quotidian practice, for the benefit of students and (ultimately) patients.

**Funding:** No

**Disclosures:** None

